# Grey Tienshan Urumqi Glacier No.1 and light-absorbing impurities

**DOI:** 10.1007/s11356-016-6182-7

**Published:** 2016-02-03

**Authors:** Jing Ming, Cunde Xiao, Feiteng Wang, Zhongqin Li, Yamin Li

**Affiliations:** National Climate Centre, China Meteorological Administration, Beijing, 100081 China; State Key Laboratory of Cryospheric Sciences, Cold and Arid Regions Environmental and Engineering Research Institute, Chinese Academy of Sciences, Lanzhou, 730000 China; Peking University Hospital, Beijing, 100871 China

**Keywords:** Black carbon (BC), Dust, Albedo, Glacier, Tienshan

## Abstract

The Tienshan Urumqi Glacier No.1 (TUG1) usually shows “grey” surfaces in summers. Besides known regional warming, what should be responsible for largely reducing its surface albedo and making it look “grey”? A field campaign was conducted on the TUG1 on a selected cloud-free day of 2013 after a snow fall at night. Fresh and aged snow samples were collected in the field, and snow densities, grain sizes, and spectral reflectances were measured. Light-absorbing impurities (LAIs) including black carbon (BC) and dust, and number concentrations and sizes of the insoluble particles (IPs) in the samples were measured in the laboratory. High temperatures in summer probably enhanced the snow ageing. During the snow ageing process, the snow density varied from 243 to 458 kg m^−3^, associated with the snow grain size varying from 290 to 2500 μm. The concentrations of LAIs in aged snow were significantly higher than those in fresh snow. Dust and BC varied from 16 ppm and 25 ppb in fresh snow to 1507 ppm and 1738 ppb in aged snow, respectively. Large albedo difference between the fresh and aged snow suggests a consequent forcing of 180 W m^−2^. Simulations under scenarios show that snow ageing, BC, and dust were responsible for 44, 25, and 7 % of the albedo reduction in the accumulation zone, respectively.

## Introduction

Mountain glaciers, different from the Arctic and Antarctic ice sheets, are geographically much closer to human settlements, such as the mid-latitude glaciers in the Alps, Caucasus, High-mountain Asia, and Southern Andes (Ming et al. 2015; Gardner et al. 2013; Zeng et al. 1984). They store water resources as snow and ice in cold seasons and release them in warm seasons, which directly adjust fresh water supplies to the lives of the surrounding people, especially to those who are living in arid regions. From this angle of view, mountain glaciers are the most important water resource in the wide arid and semi-arid regions like Central Asia.

Tien Shan Mountains, one of few areas holding most concentrated glaciers in the mid-latitudes of the northern Hemisphere, is home of nearly 16,000 glaciers (Aizen et al. 2007). More than 100 million people live on the water sourced from these glaciers, which are also called the “Water Tower of Central Asia”. The Tienshan glaciers have been shrinking since the end of Little Ice Age in the mid-nineteenth century (Sorg et al. 2012) and the shrinkage has been accelerated since the 1970s (Bolch and Marchenko 2006). A satellite gravimetric measurement revealed that the mass loss rate in the Tienshan glaciers was −5 ± 6 Gt a^−1^ (−0.32 ± 0.39 m w.e. a^−1^) for year 2003 to 2010 (Jacob et al. 2012).

The mass loss of Tienshan glaciers was primarily attributed to the rapid regional warming at a decadal rate of +0.1 to +0.2 °C since the 1970s recorded by the meteorological stations, associated with fewer precipitations contributing to drier summers (Sorg et al. 2012). However, sparse studies investigated other factors like the depositions of light-absorbing impurities (LAIs) (e.g. aerosol black carbon (BC), dust, etc.) would also induce a strong surface melting of the glaciers. This issue has been addressed by the studies regarding snow and ice of Tibet and Himalaya, northern China, and Greenland (Dumont et al. 2014; Ginot et al. 2014; Huang et al. 2011; Kaspari et al. 2014; Ming et al. 2012, 2013a, b; Qian et al. 2014; Qu et al. 2014; Wang et al. 2015; Xu et al. 2009; Zhang et al. 2015).

The Urumqi Glacier No.1 (TUG1) is one of the few Tienshan glaciers with long-term regular monitor, from which the results are annually released by the World Glacier Monitoring Service (WGMS 2012). This glacier has been experiencing a dramatic retreat since the 1980s and was separated into two branches in 1993 (Li et al. 2003, 2007). The average annual mass balance of the glacier was −286 mm w.e. over the period 1959 to 2010 (Zhang et al. 2014). The main factor inducing the shrinkage was attributed to temperature rising since the mid-1980s, simultaneously suggesting that increased precipitation was not sufficient to compromise the impact of temperature rising (Li et al. 2007).

Takeuchi and Li (2008) first suggested that dust deposited in the surface of the TUG1 substantially accounted for the shrinkage of the glacier. Xu et al. (2012) measured BC in a snow pit throughout a 2-year field observation, suggesting large variability of BC from 11 to 3000 ppb. Ming et al. (2013b) estimated that BC deposited in the glacial surface of the TUG1 could cause an annually radiative forcing of 11 W m^−2^. None of the earlier studies combined BC and dust together to investigate the comprehensive effects of the LAIs on the reduction of albedo, especially in summers, when the glacier surface experiences strong melting.

According to the observation records, the TUG1 usually displays “grey” surfaces (very low albedo) in summers, when the albedo could reach as low as 0.1 to 0.3 (Kang and Ohmura 1994). A darkened glacial surface induces strong melting consequently. Besides relatively higher temperature in summers, who could be responsible for the darkening, BC or dust? In this work, we conducted a campaign on the traverse route of the TUG1 during the summer of 2013 trying to interpret this issue partly.

## Study site and experimental methods

**Fig. 1:**
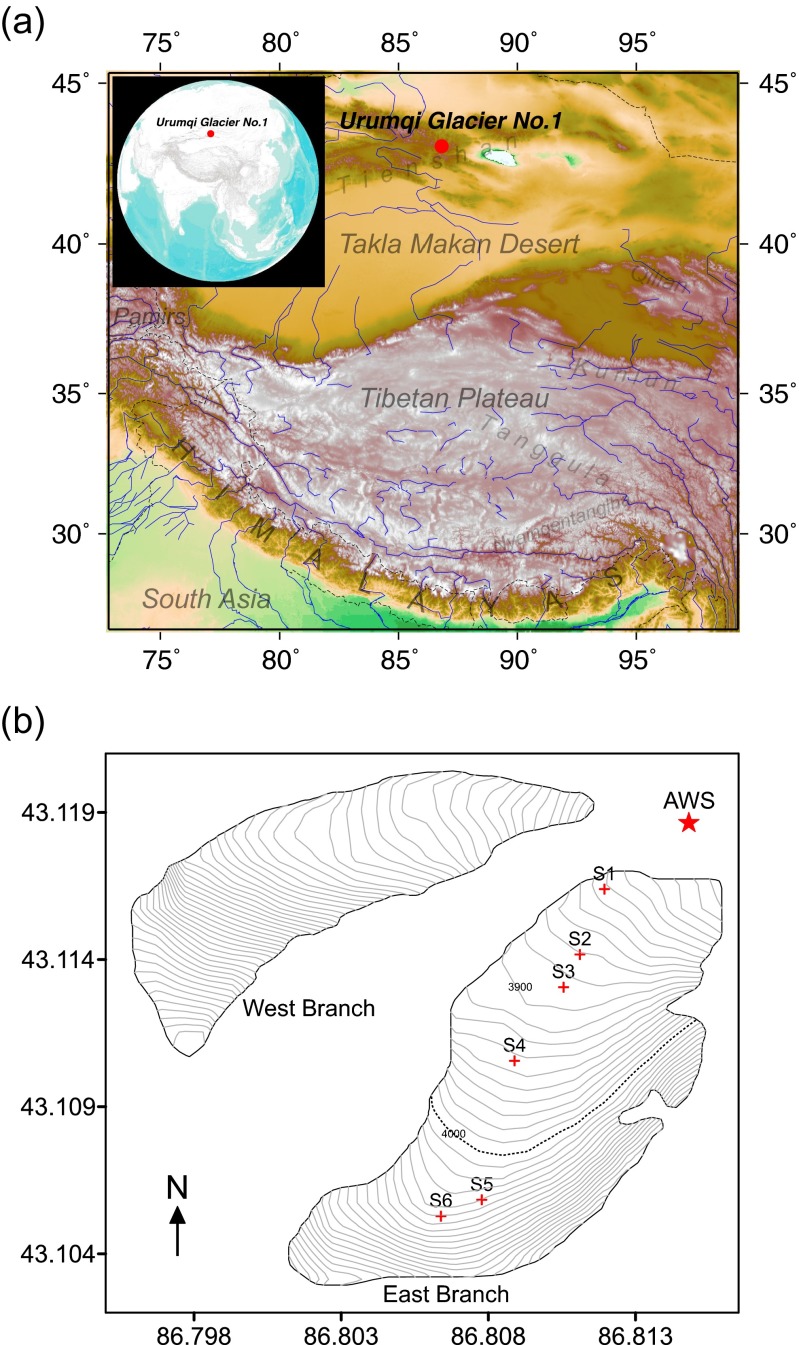
**a** Geographic map of the Urumqi Glacier No.1 (TUG1), and **b** study map of the Urumqi Glacier No.1, where sampling sites, elevation contours, and AWS are shown, and the *dashed* is the traditional ELA line

**Fig. 2:**
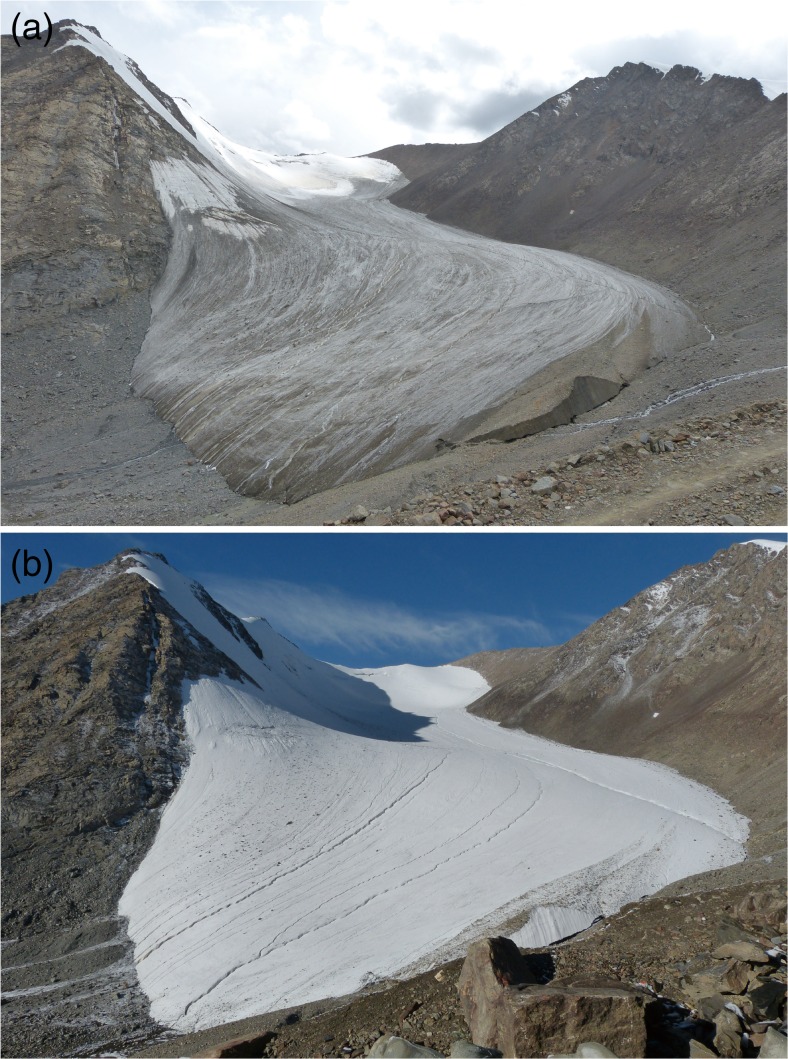
Pictures of the TUG1 taken on **a** 9th and **b** 10th August of 2013

The Tienshan Urumqi Glacier No.1 (43.10°N, 86.82°E), in the eastern Tienshan mountain (Fig. 1), is the best-monitored glacier in China (WGMS 2012). On 10 August 2013, a fine day with little cloud amount (Table 1) after a snow fall at night, we conducted a field snow sampling and observations in the traverse route of the eastern branch of the TUG1. Figure 2 describes the usual and after-snowfall looks of the TUG1 in the summer. The snow depth on the land surface was 5 to 10 cm due to the snowfall checked on the 10th morning, and the precipitation amount on the 9th to 10th was 35 mm w.e. recorded by an automatic weather station (AWS) situated near the terminal of the TUG1 (Fig. 1b).

**Table 1:** The sampling sites, field measurements, and the BC and dust and insoluble particles in snow samples

Site code	Long (°E)	Lati (°N)	Alti (m)	Cloud amount (10 = 100 %)	Sample type	Density (kg m^−3^)	Sample code	Snow grain median size (mm)	Sample volume (ml)	BC (μg)	Dust (mg)	BC (ppb)	Dust (ppm)	Particle (×10^5^ ml^−1^)	Mean particle size (μm)
S1	86.8119	43.1164	3845	2	Fresh snow	239	1-1	0.2	39.0	2.45	1.51	63	39	n/a	n/a
						171	1-2		28.0	1.74	1.07	62	38	0.72	1.35
						191	1-3		26.0	0.85	0.53	33	20	1.41	1.41
							1-4		31.5	1.54	1.59	49	50	1.88	1.52
							1-5		39.0	1.14	1.40	29	36	1.04	1.27
S2	86.8111	43.1142	3881	1	Fresh snow	188	2-1	0.35	38.0	0.96	0.61	25	16	1.36	1.48
						135	2-2		35.5	1.63	1.62	46	46	1.73	1.30
						180	2-3		45.5	1.34	0.56	30	12	2.87	1.21
							2-4		45.0	0.75	0.54	17	12	1.37	1.58
							2-5		50.0	0.97	0.46	19	9	1.05	1.31
S3	86.8106	43.1131	3893	3	Fresh snow	192	3-1	0.275	52.5	1.58	0.60	30	12	1.00	1.33
						213	3-2		54.0	0.88	0.59	16	11	0.94	1.81
						216.5	3-3		51.5	1.22	0.58	24	11	0.74	2.20
							3-4		53.0	0.82	1.16	16	22	1.08	1.66
							3-5		58.0	1.79	0.88	31	15	0.92	1.58
					Aged snow	n/a	n/a	n/a				n/a	n/a	n/a	n/a
S4	86.8089	43.1106	3934	3	Fresh snow	228.5	4-1	0.35	58.0	1.34	1.43	23	25	1.75	1.29
						215.5	4-2		52.0	1.54	0.60	30	12	1.62	1.42
						203	4-3		47.0	0.90	1.48	19	31	1.13	1.38
							4-4		64.0	1.80	0.53	28	8	1.71	1.43
							4-5		57.0	0.88	0.32	15	6	1.53	1.40
					Aged snow	n/a	4-1*	n/a	40.0	n/a	206.83	n/a	5171	2.88	3.81
						n/a	4-2*		41.0	n/a	59.48	n/a	1451	98.59	1.80
						n/a	4-3*		31.5	n/a	71.71	n/a	2277	65.12	1.93
							4-4*		34.0	n/a	206.16	n/a	6063	259.40	1.92
							4-5*		45.5	n/a	226.04	n/a	4968	45.21	1.59
S5	86.8078	43.1058	4040	1	Fresh snow	260	5-1	0.2	41.5	1.18	0.31	28	8	2.86	1.36
						301.5	5-2		65.0	1.84	1.10	28	17	2.71	1.23
						261.5	5-3		51.5	1.76	0.79	34	15	1.78	1.43
							5-4		66.5	1.45	0.71	22	11	2.22	1.38
							5-5		54.0	1.15	0.69	21	13	1.16	1.67
					Aged snow	427.5	5-1*	3	61.0	41.69	2.39	683	39	8.20	1.79
						455.5	5-2*		69.5	26.39	1.95	380	28	4.50	1.68
						492	5-3*		55.0	71.12	4.27	1293	78	3.98	1.99
							5-4*		43.5	178.07	7.68	4093	177	5.93	2.24
							5-5*		59.0	132.10	7.03	2239	119	5.55	2.31
S6	86.8064	43.1053	4050	0	Fresh snow	378.5	6-1	0.275	51.5	1.16	0.66	23	13	1.65	1.34
						344.5	6-2		61.0	1.60	0.91	26	15	1.92	1.27
						319.5	6-3		61.7	1.82	1.06	29	17	1.35	1.33
							6-4		56.7	1.53	1.00	27	18	1.98	1.34
							6-5		53.5	1.50	1.12	28	21	1.48	1.39
					Aged snow	446	6-1*	2	90.5	n/a	40.76	n/a	450	2.86	2.69
						440.5	6-2*		85.5	n/a	21.83	n/a	255	4.69	2.68
						495	6-3*		84.0	n/a	16.68	n/a	199	2.85	2.95
							6-4*		90.0	n/a	42.71	n/a	475	4.06	2.73
							6-5*		90.0	n/a	76.41	n/a	849	2.48	3.72

### Snow sampling, and the measurements of snow density and grain size

Snow samples were collected at six sites (S1 to S6) from the terminal to the accumulation zone (Fig. 1b). The surface fresh snow was about 5–10 cm in depth; the depth of the aged snow beneath surface was not directly measured here and was reported to be usually over 1 m by a multi-year snow-pit investigation (Xu et al. 2012). The distance between two closest sites was from 100 to 400 m. Aged snow completely covered the surface of the glacier on the 9th (Fig. 2a). Five fresh snow samples were collected from the same surface layer at each site, and then the upper fresh snow was scraped off using a stainless scoop and left a fresh-snow-free area of around 1 m^2^. The distinct colours and hardness feeling of aged and fresh snow may guarantee the complete removal of the fresh snow away from the top of aged snow. The aged snow was exposed and five samples were collected at each site from S4 to S6 (Table 1). All the samples were stored in pre-cleaned HDPE bottles and kept frozen until submitting to instrument for analysis. Snow densities were measured for both fresh and aged snow using an electrical scale (±1 g) and a 200-ml steel-wedge container. Snow grain sizes were measured using a ×25 lens with the precision of 0.02 mm.

### The measurement of snow surface reflectance and calculation of broadband albedo

At each site, snow surface spectral reflectance from visible to near-infrared (350–1050 nm) wavelengths was measured using a portable spectroradiometer (MS-720, Eiko Seiki, Japan). The optical measurements followed the method used by Takeuchi and Li (2008). The optical sensor of MS-720 was held and fixed at a height of 20 cm above the snow surface in the nadir-viewing position, allowing a measuring spot of 8.9 cm in diameter on snow. The optical measurement at each site was conducted randomly three times both for fresh and aged snow surfaces. Before and after the measurement at a specific site, the surface of a white reference panel that is nearly 100 % reflective and diffuse was measured by MS-720 to get the incoming irradiance. The spectral reflectance of the sites was obtained by dividing snow surface irradiance by the irradiance acquired from the reference panel. The mean of the three surface measurements at a given site is the average reflectance at that site. And the broadband albedo of a specific surface was calculated as the sum of the reflective irradiance at all spectral wavelengths divided by the sum of the incoming irradiance.

The uncertainty of measuring the albedos of fresh and aged snow remains here. For optically thin snow, the albedo of snowpack will be influenced by the albedo of underlying ground. In our work, aged snow is beneath surface fresh snow, and beneath aged snow is the glacier ice. This has been taken into account when simulating the albedos of different snow types.

### The measurements of insoluble particle numbers and sizes in snow samples

In the laboratory, snow samples were put in room temperature and allowed to melt into liquid within 2 h. Ultrasonic bath was applied for the sample bottles for 15 min to remove insoluble particles (IPs) in samples possibly attached to the walls of the bottle (Ming et al. 2008). Then 1-ml aliquot of the liquid sample was transferred by a pipette and submitted to the Single Particle Optical Sensing system (Accusizer 780A, PPS, USA). This instrument allows a single particle in the size range of 0.5 to 400 μm to pass the laser (630 μm) to measure the sizes and numbers of the IPs in the sample (Table 1). The other liquid portions of the snow sample would be applied for the measurement of LAIs.

### The measurements of the LAIs in the snow samples

The volumes of the snow samples were measured with a graduated cylinder (±1 ml) (Table 1) and then filtered through quartz-fibre filters (25 mm). These filters were preheated for 2 h in an oven at 800 °C to eliminate any carbon contents. A hand vacuum pump was used to accelerate filtering. After filtering of each sample, the containers and filtration unit were rinsed four times with ultra-pure water to make sure transferring the carbonaceous particles to the filter. The capture of BC particles was believed to be better than 97 % (Cachier and Pertuisot 1994).

The filters would be moved into the petri-slides and set in the laminar flow cabinet to let dry. The rinsing solution after washing the blank bottles with ultrapure water would pass through clean filters for making five blank filters. Before and after filtering, the mass of the filter was measured three times with a microbalance (±1 μg), respectively. The dust loading on the sample filter was determined as the weight of the filter after filtering subtracting that before filtering.

Hydrochloric acid (2–4 %) was added into the filters to remove possible carbonates for the latter BC analysis and let dry. More detailed description of the pretreatment of the snow samples before BC analysis can be referred to the earlier studies (Ming et al. 2009, 2013a). A DRI-2001® model carbon analyser (USA) was used to measure BC content in the sample filters. This instrument is built on thermal/optical reflectance (TOR) method and following the Interagency Monitoring of Protected Visual Environments (IMPROVE) protocol (Chow et al. 2004). The sample filter is heated stepwise at 120, 250, 450, and 550 °C for organic carbon (OC) in a non-oxidizing (He) atmosphere, and at 550, 700, and 800 °C for total BC in an oxidizing atmosphere of 2 % oxygen and 98 % He. Evolved carbon is oxidized to CO_2_, and then reduced to CH_4_ detected by a flame ionization detector. The portion of BC detected at 550 °C until the laser signal returns to its initial value is assigned to pyrolyzed organic carbon (OP). BC is calculated as the residue amount by subtracting the OP from total BC.

More detailed description of the working principle of DRI-2001 can be referred to by DRI (2005). The mean BC-mass density of the five blanks is 0.41 ± 0.29 μg cm^−2^. BC loadings on the sample filters were the BC masses measured by DRI subtracting the mean mass of the blank filters. Only BC is adopted for this study, and OC is not considered here (Table 1). Unfortunately, the instrument could not measure BC in the aged snow samples collected at S4, and S6, probably due to high dust loads in the filters. Only samples from the site S5 were successfully measured for BC.

## Results and discussion

### Fresh and aged snow surfaces of the UR1

The UR1 experiences strong melting in summers, when 80 % surface usually looks “grey” (Fig. 2a). However, when snow falls occasionally, it will whiten the surface, and the “white” surface usually lasts a few days (Fig. 2b). The meteorological data recorded by the AWS show that the mean temperature at the terminal of the TUG1 in the daytime of 10th August was 8 °C. High air temperatures in the summer time enhance surface snow ageing and melting. Snow density and snow grain size are very different for fresh and aged snow. The mean snow density of fresh snow from S1 to S6 is 243 kg m^−3^, while that of aged snow from S5 to S6 is 460 kg m^−3^ (Table 1). The mean snow grain size of fresh snow is 0.29 mm, while that of aged snow (2.5 mm) is much larger. The monitoring record of the TUG1 shows that the equilibrium line altitude (ELA) of the UR1 was 4267 m in 2013, which is approximately 200 m higher than the mean of 1959–2010 (Zhang et al. 2014) and indicating that extremely strong surface melting occurs. The elevations of all sampling sites are far below the ELA of 2013. These sites are all located in strong ablation zone and fresh snow can change to aged snow easily, which would make the TUG1 look not “white” any more in a short time (Fig. 2).

### The LAIs and the IPs in fresh and aged snow

**Fig. 3:**
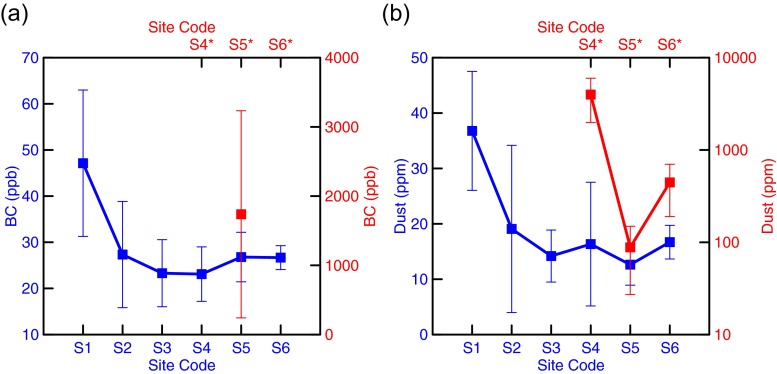
The mean concentrations of **a** BC and **b** dust in fresh (*blue line and dots*) and aged (*red line and dots*) snow at the sampling sites. *Error bars* were calculated as the standard deviations of five samples at each site

**Fig. 4:**
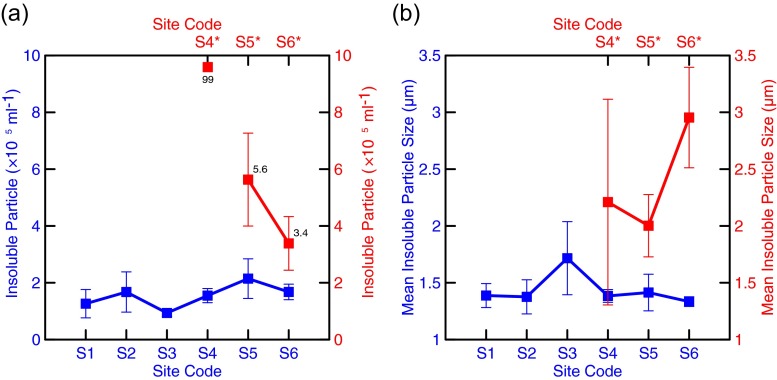
The **a** concentrations and **b** sizes of the insoluble particles in fresh (*blue line and dots*) and aged (*red line and dots*) snow at the sampling sites. *Error bars* were calculated as the standard deviations of five samples at each site

The spatial distributions of BC and dust in the sites (S1 to S6) collecting fresh snow are closely related, although their mean concentrations show large uncertainties at each site (Fig. 3). The co-variation of BC and dust in fresh snow suggests they could be well mixed after post-deposition process (Xu et al. 2012). The largest mean BC (47 ppb) in fresh snow is found at S1, in consistent with dust, of which the largest mean is 37 ppm at S1, and at the sites of S2 to S6, the concentrations of the LAIs do not show large variations, but more stable (25 ± 2 ppb for BC and 16 ± 2 ppm for dust). BC in fresh snow of TUG1 has no large difference with that measured in other typical glaciers in Tibet (Ming et al. 2013b). The spatial distributions of the LAIs in the aged-snow sites could not be discussed here in detail due to very limited data. One possibility is that the BC concentrations in the aged snow at the sites S4 and S6 should be much higher than at S5, if a similar correlation between dust and BC like in the fresh snow applies to the aged snow. The LAIs in aged snow are generally 1 to 2 orders of magnitude higher than those in fresh snow. The BC of aged snow at S5 is as high as 1738 ppb with large uncertainty (Fig. 3a), and the dust of aged snow varies from 88 ppm at S5 to nearly 4000 ppm at S4 (Fig. 3b). Variability of BC from fresh to aged snow here is similar to that measured by Xu et al. (2012). The mean of the IP numbers in fresh snow is (1.54 ± 0.41) × 10^5^ ml^−1^, showing smaller variation than that in aged snow. The IP number can reach 99 × 10^5^ ml^−1^ in aged snow at S4 (Fig. 4a). The mean IP size in fresh snow is 1.4 μm, while that in aged snow is 1-μm larger (Fig. 4b). The number-and-size variations of IP associated with BC and dust in fresh and aged snow imply IPs including BC and dust could be aggregated during the post-depositional process and darken the surface of the UR1.

### Surface reflectances and broadband albedos of fresh and aged snow and estimated forcing

**Fig. 5:**
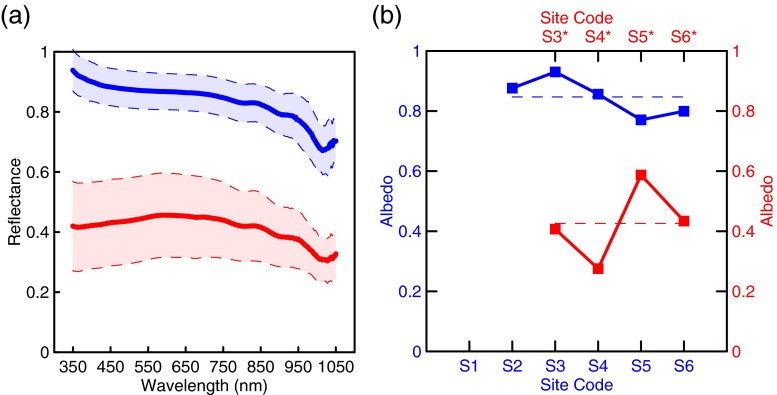
**a** The mean spectral reflectances of the fresh (*blue*) and aged (*red*) snow surfaces with one ± *σ*, and **b** the broadband albedos of the fresh (*blue*) and aged (*red*) snow surfaces

The mean spectral surface reflectances of fresh and aged snow are presented in Fig. 5a. For the fresh snow, most spectral reflectances in the wavelength range of 350–1050 nm are higher than 0.8, with no significant differences from that measured for the newly fallen snow pack at Tibet (Ming et al. 2013a), while the reflectances of the aged snow drop dramatically and vary around 0.4 at the visible and infrared wavelengths. The mean broadband albedos of the fresh and aged snow are 0.85 and 0.43, respectively, showing large differences (Fig. 5b). Roughly, higher sites have larger albedos (Fig. 5b). For fresh snow, the mean broadband albedos show smaller variability (Fig. 5a).

**Fig. 6:**
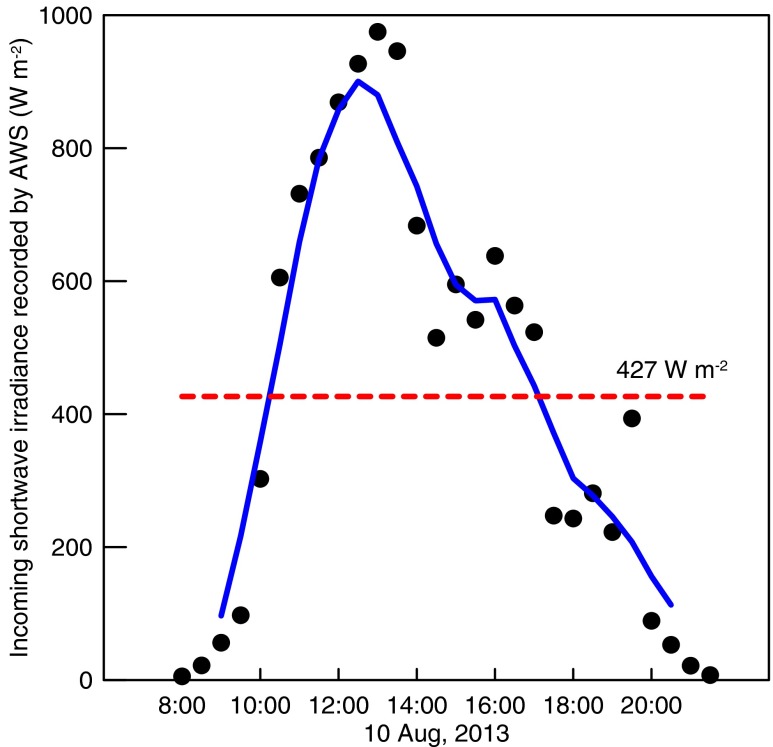
The downward solar irradiance measured by the AWS, where the *black dots* are the half-hour measurements, the *blue line* is the running average, and the *red dashed* is the mean

Downward shortwave radiation data recorded by the AWS at the terminal of the TUG1 (Fig. 1b) can be used to calculate the surface budget of solar radiation. For the enhanced directly reflecting and diffusing effect in a valley glacier, true downward solar radiation may be even stronger. On 10th August, the downward solar radiation varied between a few watts per square metre early in the morning and late in the night and nearly 1000 W m^−2^ peaking at noon (Fig. 6). In the surface of fresh snow, the net flux of solar radiation was 64 W m^−2^, taking 427 W m^−2^ as the mean incoming radiation. However, for aged snow, the net flux is as high as 243 W m^−2^. The total forcing caused by the transformation from the fresh snow to the aged could be 180 W m^−2^, presuming that the TUG1 on 10th would be evolved to the grey surface on 9th in a few days.

### Simulating the impacts of BC and dust on the snow albedo

The process of a “white” glacier turning into “grey” glacier is associated with the evolution of fresh snow to aged snow and the aggregations of the IPs including dust and BC. The online SNICAR model was developed by Flanner et al. (2007) and commonly used in simulating the impacts of LAIs on snow-and-ice albedos (Hadley and Kirchstetter 2012; McConnell et al. 2007; Qu et al. 2014). Basically, the model utilizes a two-stream radiative transfer method (Toon et al. 1989), assembles input parameters (e.g. radiation condition, surface spectral distribution, snow grain effective radius, black carbon and dust, etc.) in an online platform (http://snow.engin.umich.edu/), and can be easily operated. More details can be referred to from Flanner et al. (2007). Snow ageing (snow density increases and grain size grows) due to warming, and BC and dust aggregations were considered to be three main factors impacting surface albedo. Here, the model is applied to calculate the albedo of the fresh snow, aged snow, and other three presumed surface conditions of the TUG1. We only made a case study specific for site S5, due to the incompleteness of the dataset obtained at other sites needed for simulating the albedo reduction caused by LIAs. The snow-pack depth was set to be 1 m, according to an earlier study (Xu et al. 2012). Other different parameters needed for the fresh and aged snow were listed in Table 2.

**Table 2:** The parameters used in the albedo simulation for fresh and aged snow and other designed scenarios at site 5

Snow types and scenarios	Surface fresh snow at S5	Beneath aged snow at S5*	Snow ageing excluding BC and dust aggregation	Fresh snow + dust aggregation excluding snow ageing and BC aggregation	Fresh snow + BC aggregation excluding snow ageing and dust aggregation	Snow ageing + dust aggregation excluding BC aggregation	Snow ageing + BC aggregation excluding dust aggregation	Remark
Solar zenith angle (°)	37	37	37	37	37	37	37	
Snowpack depth (m)	0.1	1	1	0.1	0.1	1	1	
Snow density (kg m^−3^)	274	458	458	274	274	458	458	
Cloud amount (10 = 100 %)	1	1	1	1	1	1	1	Clear-sky for modelling
Snow grain radius (μm)	100	1500	1500	100	100	1500	1500	
Albedo of underlying ground (0.3–0.7 μm)	0.62	0.2	0.2	0.62	0.62	0.2	0.2	
Albedo of underlying ground (0.7–5.0 μm)	0.53	0.1	0.1	0.53	0.53	0.1	0.1	
Sulfate-coated BC (ppb)	27	1738	27	27	1738	27	1738	
Dust (ppm)	13	88	13	88	13	88	13	
Mean particle size (μm)	1.0–2.5	1.0–2.5	1.0–2.5	1.0–2.5	1.0–2.5	1.0–2.5	1.0–2.5	Mean sizes are 1.41 and 2.00 μm for fresh and aged snow, respectively
MAC scaling factor (experimental)	11.3	11.3	11.3	11.3	11.3	11.3	11.3	Hydrophilic BC
Simulated albedo	0.782	0.325	0.580	0.752	0.667	0.511	0.316	
Measured albedo	0.771	0.587						
Albedo reduction		0.457	0.202	0.030	0.115	0.271	0.466	
Attributable proportion (%)			44	7	25	59		

Here are some descriptions how to choose the parameters to simulate the albedo of different snow using online SNICAR model. Solar zenith angle was calculated according to the local sampling time and geographic locations. Snowpack depth was considered as 0.1 m for fresh snow and 1 m for aged snow. Snow densities were taken as measured. Clear sky and direct radiation were chosen for the very low cloud amount (∼1). We consider aged snow and glacier ice are the underlying grounds for fresh snow and aged snow. Thus, the underlying albedos of fresh and aged snow was set as 0.62 (0.3–0.7 μm) and 0.53 (0.7–5.0 μm) as measured, and 0.2 (0.3–0.7 μm) and 0.1 (0.7–5.0 μm) by Zeng et al. (1984), respectively. BC was thought to be sulfate-coated after long-distance transport (Ming et al. 2009) and to be hydrophilic with the median mass absorption cross section (MAC) of 11.3 m^2^ g^−1^ (Flanner et al. 2007). Mean particle size was set according to the measured mean size.

Simulated albedo of the fresh snow at S5 was 0.77, close to the measured value (0.78). The model did not simulate the expected albedo of aged snow at S5. The simulated albedo of the beneath aged snow was 0.33, remarkably lower than the measured (Table 2). The simulated albedo was close to that of the aged snow only considering snow ageing, possibly indicating snow ageing due to warming was the predominant to reduce the snow albedo, concealing the impacts of other LAIs during the post-deposition processes. We presumed another two independent scenarios; only dust and only BC with measured concentrations were applied to fresh snow, separately. In the condition fresh snow only with dust aggregation was considered, the albedo decreased from 0.78 to 0.75. If fresh snow only with BC aggregation was considered, the albedo decreased from 0.78 to 0.67. The effect of BC on albedo reduction exceeded that of dust. However, the reality was the measured albedo of aged snow is not the expected simulated value, but far higher than that. This possibly indicates that dust deposited on the surface of the TUG1 could offset or conceal the impact of BC on albedo, which has been similarly reported by earlier studies (Ginot et al. 2014; Kaspari et al. 2014). In general, snow ageing could account for 44 % of the total albedo reduction, dust only could be responsible for 7 % reduction, and BC only would take part in 25 % of albedo reduction at site 5, calculated from the relevant decreases in albedo derived from selected-parameter-based simulations. For being lack of more comprehensive observation data, a complete conclusion of the impacts of LAIs on the surface albedo of TUG1 glacier cannot be drawn here.

## Conclusions

Fresh and aged snow samples were collected on the east branch of the TUG1 on 10 August 2013 after a snowfall at night. Measurements including snow densities and grain sizes and spectral reflectances were made in the transect route from the terminal to the accumulation zone. High temperatures in the summer probably enhanced the surface snow ageing and melting. The snow density and grain size increased from 243 to 458 kg m^−3^ and from 290 to 2500 μm during the ageing process, along with the number and size of the IPs increase. Concentrations of dust and BC in fresh snow are 16 ppm and 25 ppb, respectively. While in aged snow beneath the fresh, their concentrations can be as high as 1507 ppm and 1738 ppb. The albedo discrepancy of fresh and aged snow can be as large as 0.40, indicating a consequent forcing of 180 W m^−2^ during the post-deposition process. At the accumulative zone, snow ageing (44 %) is the most significant factor decreasing the albedo, BC (25 %) is the next, and then dust (7 %). For the lack of dataset, the complete conclusion of impacting factors decreasing the surface albedo of TUG1 cannot be drawn at present.

## References

[CR1] Aizen VB, Aizen EM, Kuzmichonok VA (2007). Glaciers and hydrological changes in the Tien Shan: simulation and prediction. Environ Res Lett.

[CR2] Bolch T, Marchenko S (2006) Significance of glaciers, rockglaciers and ice-rich permafrost in the Northern Tien Shan as water towers under climate change conditions. Proceedings of the Workshop Assessment of Snow-Glacier and Water Resources in Asia, pp. 28–30

[CR3] Cachier H, Pertuisot M (1994). Particulate carbon in Arctic ice. Analusis.

[CR4] Chow J, Watson J, Chen L, Arnott W, Moosmuller H, Fung K (2004). Equivalence of elemental carbon by thermal/optical reflectance and transmittance with different temperature protocols. Environ Sci Technol.

[CR5] DRI (2005) DRI STANDARD OPERATING PROCEDURE: DRI model 2001 thermal/optical carbon analysis (TOR/TOT) of aerosol filter samples—method IMPROVE_A. In: Division of Atmospheric Sciences DRI (Hrsg.), Reno, NV, pp. 1–79

[CR6] Dumont M, Brun E, Picard G, Michou M, Libois Q, Petit JR, Geyer M, Morin S, Josse B (2014). Contribution of light-absorbing impurities in snow to Greenland’s darkening since 2009. Nat Geosci.

[CR7] Flanner MG, Zender CS, Randerson JT, Rasch PJ (2007). Present-day climate forcing and response from black carbon in snow. J Geophys Res.

[CR8] Gardner AS, Moholdt G, Cogley JG, Wouters B, Arendt AA, Wahr J, Berthier E, Hock R, Pfeffer WT, Kaser G, Ligtenberg SRM, Bolch T, Sharp MJ, Hagen JO, van den Broeke MR, Paul F (2013). A reconciled estimate of glacier contributions to sea level rise: 2003 to 2009. Science.

[CR9] Ginot P, Dumont M, Lim S, Patris N, Taupin JD, Wagnon P, Gilbert A, Arnaud Y, Marinoni A, Bonasoni P, Laj P (2014). A 10 year record of black carbon and dust from a Mera Peak ice core (Nepal): variability and potential impact on melting of Himalayan glaciers. Cryosphere.

[CR10] Hadley OL, Kirchstetter TW (2012). Black-carbon reduction of snow albedo. Nat Clim Chang.

[CR11] Huang J, Fu Q, Zhang W, Wang X, Zhang R, Ye H, Warren SG (2011). Dust and black carbon in seasonal snow across Northern China. Bull Am Meteorol Soc.

[CR12] Jacob T, Wahr J, Pfeffer WT, Swenson S (2012). Recent contributions of glaciers and ice caps to sea level rise. Nature.

[CR13] Kang E, Ohmura A (1994). A parameterized energy balance model of glacier melting on the Tianshan Mountain. Acta Geograph Sin.

[CR14] Kaspari S, Painter TH, Gysel M, Skiles SM, Schwikowski M (2014). Seasonal and elevational variations of black carbon and dust in snow and ice in the Solu-Khumbu, Nepal and estimated radiative forcings. Atmos Chem Phys.

[CR15] Li Z, Hang T, Jing Z, Yang H, Jiao K (2003). A summary of 40-year observed variation facts of climate and Glacier No. 1 at headwaters of Urumqi River, Tianshan, China. J Glaciol Geocryol.

[CR16] Li Z, Shen Y, Wang F, Li H, Dong Z, Wang W, Wang L (2007). Response of melting ice to climate change in the Glacier No. 1 at the headwaters of Urumqi River, Tianshan Mountain. Adv Clim Chang Res.

[CR17] McConnell J, Edwards R, Kok G, Flanner M, Zender C, Saltzman E, Banta J, Pasteris D, Carter M, Kahl J (2007). 20th-Century industrial black carbon emissions altered arctic climate forcing. Science.

[CR18] Ming J, Cachier H, Xiao C, Qin D, Kang S, Hou S, Xu J (2008). Black carbon record based on a shallow Himalayan ice core and its climatic implications. Atmos Chem Phys.

[CR19] Ming J, Xiao C, Cachier H, Qin D, Qin X, Li Z, Pu J (2009). Black carbon (BC) in the snow of glaciers in west China and its potential effects on albedos. Atmos Res.

[CR20] Ming J, Du Z, Xiao C, Xu X, Zhang D (2012). Darkening of the mid-Himalaya glaciers since 2000 and the potential causes. Environ Res Lett.

[CR21] Ming J, Wang P, Zhao S, Chen P (2013). Disturbance of light-absorbing aerosols on the albedo in a winter snowpack of Central Tibet. J Environ Sci.

[CR22] Ming J, Xiao C, Du Z, Yang X (2013). An overview of black carbon deposition in high Asia glaciers and its impacts on radiation balance. Adv Water Resour.

[CR23] Ming J, Wang Y, Du Z, Zhang T, Guo W, Xiao C, Xu X, Ding M, Zhang D, Yang W (2015) Widespread albedo decreasing and induced melting of Himalayan snow and ice in the early 21st century. PLoS One 10(6):e0126235. doi:10.1371/journal.pone.0126235 10.1371/journal.pone.0126235 PMC4454657 26039088

[CR24] Qian Y, Wang H, Zhang R, Flanner MG, Rasch PJ (2014). A sensitivity study on modeling black carbon in snow and its radiative forcing over the Arctic and Northern China. Environ Res Lett.

[CR25] Qu B, Ming J, Kang SC, Zhang GS, Li YW, Li CD, Zhao SY, Ji ZM, Cao JJ (2014). The decreasing albedo of the Zhadang glacier on western Nyainqentanglha and the role of light-absorbing impurities. Atmos Chem Phys.

[CR26] Sorg A, Bolch T, Stoffel M, Solomina O, Beniston M (2012). Climate change impacts on glaciers and runoff in Tien Shan (Central Asia). Nat Clim Chang.

[CR27] Takeuchi N, Li Z (2008). Characteristics of surface dust on Ürümqi Glacier No. 1 in the Tien Shan Mountains, China. Arct Antarct Alp Res.

[CR28] Toon OB, McKay CP, Ackerman TP, Santhanam K (1989). Rapid calculation of radiative heating rates and photodissociation rates in inhomogeneous multiple scattering atmospheres. J Geophys Res.

[CR29] Wang M, Xu B, Kaspari S, Gleixner G, Schwab VF, Zhao H, Wang H, Yao P (2015). Century-long record of black carbon in an ice core from the Eastern Pamirs: estimated contributions from biomass burning. Atmos Environ.

[CR30] WGMS (2012). Fluctuations of glaciers 2005–2010.

[CR31] Xu B, Cao J, Hansen J, Yao T, Joswia DR, Wang N, Wu G, Wang M, Zhao H, Yang W, Liu X, He J (2009). Black soot and the survival of Tibetan glaciers. Proc Natl Acad Sci U S A.

[CR32] Xu B, Cao J, Joswiak D, Liu X, Zhao H, He J (2012). Post-depositional enrichment of black soot in snow-pack and accelerated melting of Tibetan glaciers. Environ Res Lett.

[CR33] Zeng Q, Cao M, Feng X, Liang F, Chen X, Sheng W (1984) Study on spectral reflection characteristics of snow, ice and water of Northwest China. Jbxg

[CR34] Zhang G, Li Z, Wang W, Wang W (2014). Rapid decrease of observed mass balance in the Urumqi Glacier No. 1, Tianshan Mountains, central Asia. Quat Int.

[CR35] Zhang Y, Hirabayashi Y, Liu Q, Liu S (2015). Glacier runoff and its impact in a highly glacierized catchment in the southeastern Tibetan Plateau: past and future trends. J Glaciol.

